# The Use of Infrared Thermography in the Assessment of Thermal Reaction of Patients Treated with Radiotherapy after Breast-Conserving Procedures

**DOI:** 10.3390/ijerph192114187

**Published:** 2022-10-30

**Authors:** Dominika Plaza, Agnieszka Baic, Barbara Lange, Łukasz Michalecki, Krzysztof Ślosarek, Agata Stanek, Armand Cholewka

**Affiliations:** 1 Radiotherapy Planning Department, Maria Skłodowska-Curie National Research Institute of Oncology Gliwice Branch, Wybrzeze Armii Krajowej Street 15, 44-102 Gliwice, Poland; 2 Faculty of Science and Technology, University of Silesia, 75 Pułku Piechoty 1A, 41-500 Chorzów, Poland; 3 IIIrd Radiotherapy and Chemotherapy Department, Maria Skłodowska-Curie National Research Institute of Oncology Gliwice Branch, Wybrzeze Armii Krajowej Street 15, 44-102 Gliwice, Poland; 4 Department of Radiation Oncology, University Clinical Center, Medical University of Silesia, Ceglana Street 35, 40-514 Katowice, Poland; 5 Department and Clinic of Internal Medicine, Angiology and Physical Medicine, Faculty of Medical Sciences in Zabrze, Medical University of Silesia, Batorego 15 St., 41-902 Bytom, Poland

**Keywords:** infrared thermography, radiotherapy, isodoses, isotherms, breast cancer

## Abstract

The aim of the study was to assess the usefulness of the infrared thermography method in the assessment of the body’s thermal reaction in patients during radiotherapy. Studies have shown how the temperature distribution changes with the dose of radiation used in each treatment week. Fifty-four patients participated in the study. The control group consisted of healthy patients, among whom the difference in mean temperatures between the breasts was checked. The study group included patients after conserving procedures qualified for radiotherapy. Measurements were taken and analyzed for each patient during each week of treatment. The target area (marked by a doctor) and the 30 Gy, 20 Gy and 10 Gy isodoses (generated from the treatment plan) were transferred on thermograms and then analyzed. This way of defining the observed areas is the most accurate and individually adjusted to each treated patient. The analysis showed an increase in temperature in the tested body surface areas, whereas the highest average temperature value was achieved during the third week of treatment. The observations may be used to evaluate the skin reaction from radiation; however, they require further studies and new quantitative parameters.

## 1. Introduction

Breast cancer is the most common female cancer [1]. As a result, screening tests aimed at looking for cancer in people without symptoms of the disease have become very popular in recent years [2,3,4]. They lead to reduced mortality and allow for more effective treatment. Patient education related to self-monitoring of the breasts is also important, as it allows for the early detection of palpable breast tumors. An additional factor increasing the chances of recovery is the development of medicine [5,6]. Treatment is individually tailored to the patient and is often based on combination therapies: chemotherapy, hormone therapy, immunotherapy and radiotherapy. Patients diagnosed with breast cancer may be eligible for radiation therapy. This method is becoming more and more popular. It is an integral part of combination therapy. It is currently used at all levels of advancement. Radiotherapy after conserving procedures is a necessary complement to the surgical procedure [7].

Before starting radiotherapy, the patients undergo standard preparatory procedures. These include the fabrication of a thermoplastic mask or a mattress, which during treatment on a therapeutic apparatus, allows for reconstruction of the same patient’s position and minimizes his or her movements. The next step is to perform a tomographic examination, which later on allows the doctor to determine the planning target volume (PTV) [8,9]. Critical structures are also drawn. For breast cancer, these are: lungs, spinal canal, heart and liver. The physicist uses the treatment planning system (TPS) to execute the treatment plan. The National Institute of Oncology uses the Eclipse treatment planning system from Varian Medical Systems. The physicist chooses the irradiation technique, power, geometry of the therapeutic beams and their number. The radiotherapist gives the values of the fractional dose (during one treatment) and the total dose. In the case of breast cancer, the standard fractional dose is 2–2.5 Gy, and the total dose is 45–50 Gy [9]. The experience of a medical physicist allows for creating the most beneficial plan for the patient, where the PTV area receives the desired dose and critical organs are protected. Thanks to the enormous development of irradiation techniques and the expansion of the treatment planning system, the plans become more and more precise. Several years ago, the shape of the irradiation field made it possible to recreate simple shapes, which were not perfectly suited to the tumor and the surrounding organs. Thanks to the use of a multi-leaf collimator (MLC), the distribution of isodoses adapts to the shape of the irradiated target defined by the doctor, and what is more, it allows for even better protection against the radiation of healthy organs located in the vicinity. MLCs consist of two opposite collimators and are divided into independently moving leaves. The most common techniques for treating breast cancer in Gliwice are intensity-modulated radiation therapy (IMRT) and volumetric-modulated arc therapy (VMAT) [10,11]. The difference between these techniques is due to the position of the accelerator head. In the case of the first method, it is constant, whereas in the VMAT technique, the accelerator head rotates with modulated rotational speed and the dose rate changes. Once the treatment plan is completed, it is approved. The physician and the person carrying out the plan assess its physical parameters [12].

Each treatment plan is presented to a medical council. It consists of the head of the organizational unit, attending physicians, trainee doctors and a physicist. Before starting the treatment, the verification physicist checks whether the plan has been performed correctly. Such double checking reduces the probability of the error. Only after such preparation is performed, the patient begins the treatment. The patient’s position on the therapy table must be accepted by the doctor before the first radiotherapy. It is also verified against each fraction, most often with X-rays. The correct position of the patient is extremely important for the tumor to receive the planned dose and to spare healthy organs. As a standard, the patient is treated five days a week. They meet their attending physician once a week to assess their health [13,14,15,16].

The temperature distribution in the human body depends on the metabolic rate of a given organ and the activity of the vascular system. It does not determine a constant temperature in the whole human body, but can determine the temperature of chosen areas of the body. Hence, significant deviations from the prevailing temperature in a given area may indicate abnormalities, functional changes, acceleration of metabolism, changes in blood supply in organs located under the skin of that area. Temperature differences are evident every time when there are changes in blood supply [17,18,19]. They may indicate local inflammation, ischemia or increased blood supply. Then, based on the analysis of the heat map, the size of the area of increased or in some cases lowered temperature is determined, which deviates from normal physiology. If possible, the thermal asymmetry between the test site on the body with altered temperature and the healthy organ on the opposite side of the body should be taken into account (in the case of comparing the temperature on the surface of each of them). In addition, the temperature change may also be caused by external factors that the patient undergoes during treatment, e.g., with ionizing radiation during radiotherapy [20,21]. Thermal imaging allows to register the infrared radiation emitted by the surface of the body and gives the possibility to obtain the thermal body map [22,23,24]. The advantage of this type of imaging is the fact that it is a non-invasive method, which is why it has been successfully used in medicine for several dozen years. Its benefits are also noticed and used by specialists in biomedical engineering, medical physics and physiotherapy [25]. It is mainly a supplementary method to diagnostic tests, but thanks to the availability of more and more accurate devices that allow for the analysis of the temperature distributions of the body surface and the development of new methods of analyzing the obtained thermal images, it can be regarded as a developing method with great potential. This is evidenced by numerous scientific publications and available literature [22,23,24,25,26,27,28,29,30,31,32,33,34,35,36,37,38,39,40]. Thermovision allows you to monitor the effectiveness of osteoporosis therapy; it is used in the diagnosis of diseases of the temporomandibular joints, in rheumatology, in diagnosing diseases of the motor organs and to determine the extent of burns and skin diseases [25,26,27,28,29,30]. For many years, attempts have been made to use thermographic examinations as tests supporting the diagnosis of breast cancer [31,32,33,34,35,36,37]. These studies provide additional information on not only the localization of pathology, but also changes in metabolic activity [38,39,40,41,42].

The aim of the study was to assess the usefulness of the infrared thermography method in the assessment of the body’s thermal reaction in patients during radiotherapy [43,44,45]. Studies have shown how the temperature distribution changes with the dose of radiation used in each treatment week [46]. The innovative idea in this paper is to compare isodoses with isotherms. The additional aim of the work was to check in which of the analyzed areas and what time after the commencement of treatment, the temperature values showed statistically significant differences.

## 2. Materials and Methods

The project “Use of thermal imaging in cancer radiotherapy” was approved by the Bioethics Committee at the Center of Oncology—Maria Skłodowska-Curie Institute (the current name is the Maria Skłodowska—Curie National Research Institute of Oncology (NIO-PIB) in Warsaw on 6 October 2016 (Number 38/2016)).

Fifty-four patients participated in the study. The control group consisted of twenty-seven healthy patients. The mean age was 53 ± 10 years, 1.64 ± 0.05 m height and 72.11 ± 10.70 kg weight. The BMI of the patients was calculated, and the mean value is 26.92 ± 4.13 kg/m^2^. The research group included twenty-seven patients who were qualified for the treatment of breast cancer with radiotherapy by the decision of a medical consultation. The mean age of this group was 57 ± 13 years, 1.62 ± 0.05 m height and 67.44 ± 10.83 kg weight. The BMI of the patients was calculated and the mean value is 25.71 ± 4.58 kg/m^2^.

The research was carried out at the Maria Skłodowska—Curie National Research Institute of Oncology branch in Gliwice in cooperation with the Institute of Biomedical Engineering, University of Silesia. The room in which the tests were carried out had a constant temperature of 22 °C ± 1 °C with humidity ranging from 40% to 45%. Measurements were made with the FLIR System E60 thermal imaging camera (detector resolution 320 × 240 pixels, thermal sensitivity 0.05 K) [47]. Before making the decision to participate in the research, the patients were thoroughly informed about its course by specially trained personnel. Additionally, each participant received a “Patient Information Form”. The participants did not bear any costs and did not receive any financial remuneration. After obtaining written consent for participation, each patient completed a questionnaire. It consisted of several parts and included: information about the patient (age, height, weight, length of the menstrual cycle), history of possible breast disease or comorbidities and their course, pregnancy and lactation (number of pregnancies, births and duration of breastfeeding)). All information collected during the audit was assigned an appropriate number and stored in accordance with the applicable legal status. The criteria necessary for inclusion in the study were: no alcohol consumption for at least two hours before the study, no vigorous physical exertion on the day of the examination, no infection with a body temperature exceeding 37.5 °C and no sunbathing on the day of the study. Then the patients took off the top garments and stayed at rest for twenty minutes in order to acclimatize to the temperature of the room. After this time, the main part of the study registration of thermograms took place. A constant distance from the patient was applied, which was two meters [23,24,25]. The examinations were always performed in a standing position with arms raised. Direct, left and right thermograms were made for each patient [45,46]. It was analyzed whether there is a temperature difference between the breast area of healthy patients and the breasts of patients undergoing radiotherapy.

**Figure 1:**
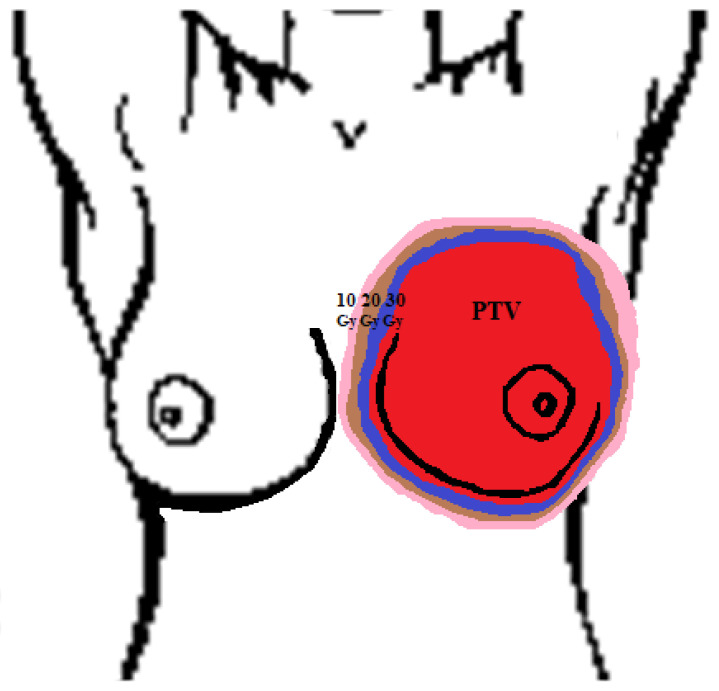
Exemplary isodose drawing scheme generated from the individual treatment plan for each patient—PTV area (red), 30 Gy (navy blue), 20 Gy (brown) and 10 Gy (pink).

The research group underwent tests before starting radiotherapy, and then after each week of the treatment. Participation in the study was not associated with exposure to additional harmful factors. The methods used were non-invasive, and their advantage is that they did not cause any side effects and could be repeated many times. The tests performed did not affect the starting date and duration of the treatment for any patients. Meetings with each patient were always held before administering the fractional dose, each time in a properly prepared room. In addition, the well-being of the respondents was checked, the condition of the skin in the irradiated area was assessed and the occurrence of possible side effects was noted. The spatial distribution of the dose in the patient’s body is presented in the form of isodoses. These are lines connecting points with the same dose values. Isodoses for the target and for the values of 30 Gy, 20 Gy and 10 Gy were placed on the thermogram made for the patient. Their size depends on the size of the irradiated area (determined by the doctor) and the implementation of the treatment plan. Each treatment plan is designed for the patient individually, and it will be different for each of them. Therefore, this method of controlling the irradiation site is the most accurate since the areas are determined individually for each patient depending on the treatment plan. Both the control group and the patients had the same areas of the body surface tested for which the thermal imaging measurements were performed. The method of thermogram analysis for healthy patients was different from that used for patients treated with radiotherapy. In healthy patients, one measurement was performed and the difference between breasts (right and left) was checked. In the research group (in treated patients), PTV and isodose areas were generated from the treatment plan and transferred to thermograms. Five measurements were made before radiotherapy and in each week of treatment, and their changes over time were analyzed. The scheme of drawn isodoses is presented in Figure 1.

Statistical analysis was performed using the STATISTICA 10 program, which contains a complete set of statistical tools and methods for comprehensive development and graphical presentation of the results of clinical trials. The confidence interval was 0.95. For each analysis, the Shapiro–Wilk test was performed to check the type of distribution of measurable features, and it was checked whether the distribution of the variables was normal. Moreover, the homogeneity of variances was checked. On the basis of homogeneity of variances and normality of variable distribution, it was decided to perform parametric tests (including the Student’s *t*-test for dependent groups in the case of, for example, the analysis of temperature changes over time, or independently examining the differences in temperature between the groups of healthy and treated patients). When the essential assumptions for parametric tests were not met, non-parametric tests were used (including the Wilcoxon test). Equality of the studied groups was taken care of. The level of significance was *p* < 0.05. The results were presented using graph boxes.

## 3. Results

The thermograms of an exemplary patient are presented below.

**Figure 2:**
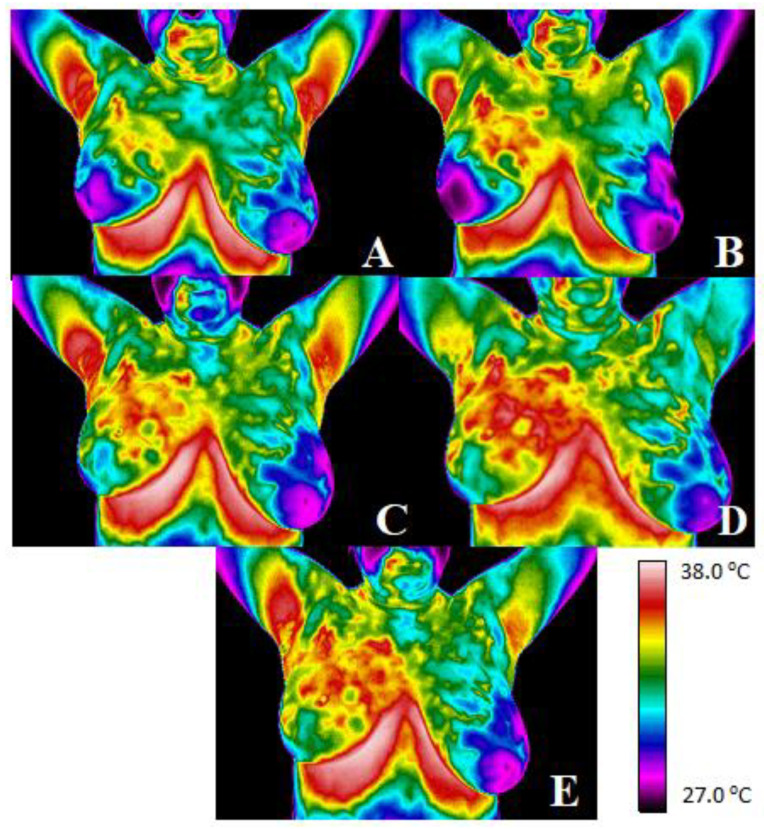
Thermograms of an exemplary patient taken before radiotherapy (thermogram (**A**)), after the first week of treatment (thermogram (**B**)), after the second week of treatment (thermogram (**C**)), after the third week of treatment (thermogram (**D**)) and after the fourth week of treatment (thermogram (**E**)).

Pictures from Figure 2 show thermograms of an exemplary patient taken before radiotherapy (thermogram, Figure 2A), after the first week of treatment (thermogram, Figure 2B), after the second week of treatment (thermogram Figure 2C), after the third week of treatment (thermogram, Figure 2D) and after the fourth week of treatment (thermogram, Figure 2E). Thermograms are presented with a color map to easily show how the patient’s temperature distribution changes during radiation therapy. One can see that the thermal asymmetry before breast-conserving treatment is rarely visible but increases with each week of the treatment. Similar increases were observed when it comes to every single area analyzed every week after irradiation. The mean temperature value in the defined PTV area obtained before treatment was 33 °C, and after the first week of treatment, the temperature increased to 33.4 °C. In the second week of treatment, it increased by 0.6 °C and amounted to 34 °C. The greatest increase was recorded in the third week of treatment (thermogram, Figure 2D), and it was 1.9 °C when compared to the test before radiotherapy. The temperature in the fourth week was lower than in the third and amounted to 34.5 °C. However, its value was still higher than before radiotherapy.

Table 1 presents temperature changes in the PTV area for 10 exemplary patients in the study group of patients during radiotherapy. The differences between the third week of treatment and the pre-radiotherapy temperature values were calculated and collected.

**Figure 3:**
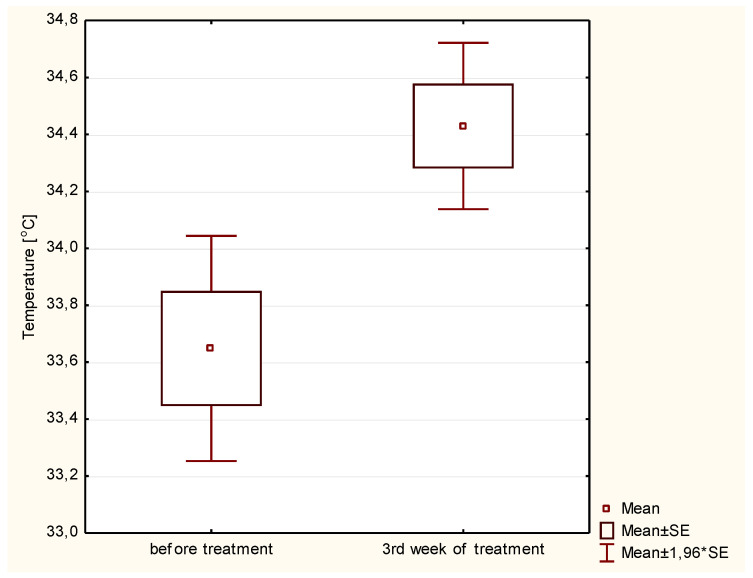
Average temperature of patients in the PTV area before and after 3 weeks of radiotherapy.

Statistical tests were performed to provide a better insight into the differences in body temperature observed during the therapy. Figure 3 shows the mean temperature value before treatment and after three weeks of treatment in the PTV area. Compared to the pre-radiotherapy thermograms and thermograms in the third week of treatment, the mean temperature increase in the PTV area was 0.78 °C. These differences are statistically significant (*p* < 0.005).

Measurements of the temperature distribution before radiotherapy and in each treatment week were verified in the target area, where 30 Gy, 20 Gy and 10 Gy isodoses were applied.

**Figure 4:**
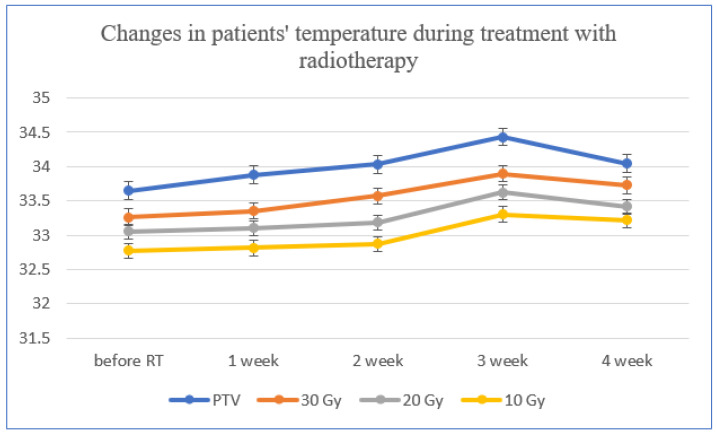
Average temperatures in the target area (PTV) and the isodose area of 30 Gy, 20 Gy and 10 Gy for patients undergoing radiotherapy treatment.

Figure 4 shows that with the passage of consecutive weeks of the treatment, both the target average temperature and the analyzed areas of isodoses temperature increased. Patients were given fractional doses five times a week; therefore, the dose they received was higher in each week of the study. The highest mean temperature was recorded in the third week of the treatment, which seems to be correlated with the appearance of skin erythema (which usually is seen within the third week of the treatment). Such a phenomenon corresponds with data available in the literature [43,44,45]. The highest temperature increase was obtained in the PTV area where the highest dose was administered. A deeper analysis of the results showed for which area (PTV, 30 Gy, 20 Gy and 10 Gy) and after what period of time (in which week of treatment) the temperature changes will be statistically significant compared to the values measured before the initiation of radiotherapy. In the case of the PTV area, after the first week of treatment, the differences become statistically significant, whereas for the 30 Gy area, statistically significant differences appear in the second week of treatment. For the area of 20 Gy and 10 Gy, statistically significant differences are visible only in the third week of treatment, compared to the temperature measured before radiotherapy.

The difference in average temperatures in the analyzed areas is presented in Table 2 When we compare the isodoses with temperature in corresponding areas, it can be seen the skin temperature change is directly proportional to the magnitude of the administered dose. The highest temperature rise was recorded in the third week of treatment. When comparing the PTV and 30 Gy area, statistically significant differences were found, and the statistical significance was valued at the level (*p* < 0.005). The mean temperature value for all patients in the PTV area amounted to 34.43 °C, and in the 30 Gy area it was 33.90 °C. There were also statistically significant differences for the mean temperatures in the PTV area and 20 Gy (where the average temperature was 33.63 °C). The performed Student’s *t*-test showed that (*p* < 0.005). Additionally, when comparing the temperatures in the area of PTV and 10 Gy (where the average temperature was 33.30 °C), statistically significant differences were also found (*p* < 0.005). Such results confirm the dependence between dose and average temperature in respective areas.

Careful analysis of the observed correlation provides us with additional information. It seems that in case of an error in the implementation of the treatment plan or incorrect positioning of the patient on the apparatus, a difference in the temperature map in the target area may be observed. This may lead to the conclusion that thermal imaging may be helpful as a non-invasive method of assessing the temperature effects of radiotherapy treatment.

**Figure 5:**
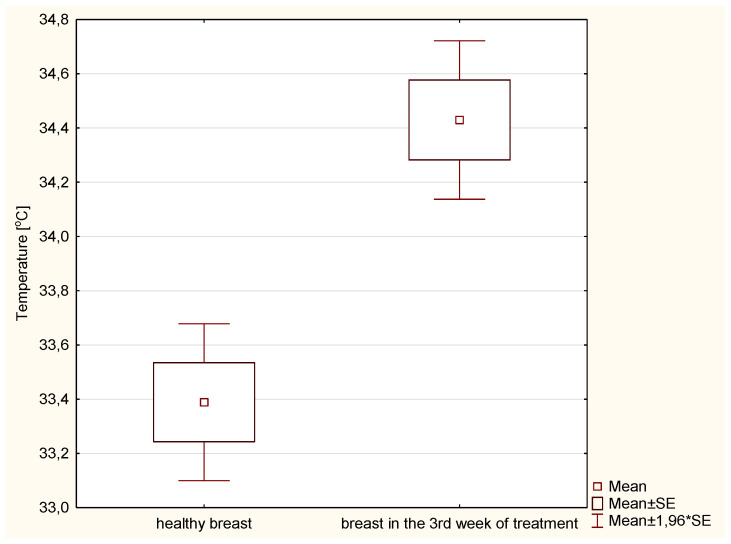
Average temperatures in the third week of treatment in the PTV area and for a healthy breast.

Additionally, the mean temperature between the treated area in the third treatment week and the healthy breast was compared. The average temperature in the designated target area was 34.43 °C, while in the healthy breast it was 33.39 °C, as shown in Figure 5. These differences are statistically significant (*p* < 0.005).

**Figure 6:**
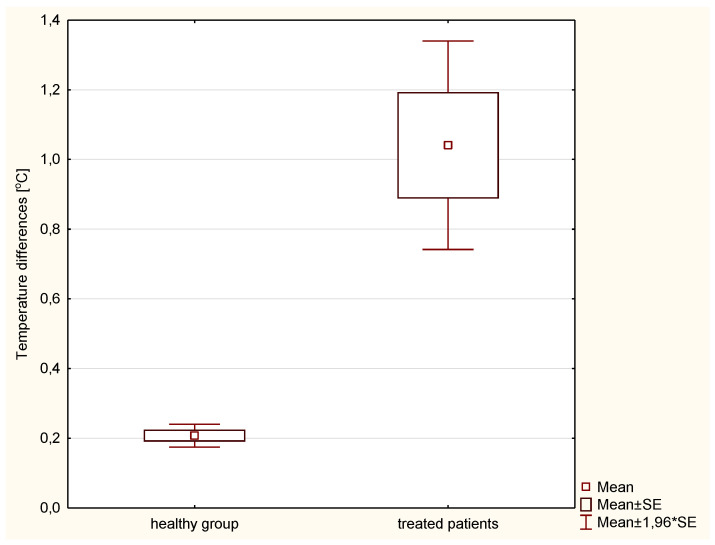
Differences in mean temperatures between breasts (right and left) in healthy patients and differences in mean temperatures between PTV and healthy breasts in patients in the third week of treatment.

For healthy women, the mean temperature values for the left breast were 32.86 °C, and for the right breast 32.81 °C. The mean temperature difference between the two areas was 0.21 °C. These differences are not statistically significant (*p* > 0.005). No significant thermal asymmetry between breasts was observed in the group of healthy women, which is also consistent with the literature [36,37,38,39,40,41]. What has to be mentioned is that the average difference in mean values of temperatures between the healthy (non-treated) breast and the PTV area was at the level of 1.04 °C five times higher than in healthy patients. The comparison was made in the third week of the treatment, where the observed temperature rise was the highest. Observed differences are statistically significant (*p* < 0.005). and results are presented in Figure 6. In patients before radiotherapy, the difference between the PTV-treated area and the healthy breast was 0.3 °C. This value is higher than in healthy women, but the differences are not statistically significant. The temperature of the healthy breast did not change during time significantly. On the other hand, before radiotherapy, the temperature of the untreated breast was 33.35 °C, while in the third week of treatment it was 33.39 °C.

## 4. Discussion

The limitations of this method are certainly the exclusion criteria for participation in the examination, which include: consumption of alcohol, stimulants and smoking for at least two hours before the examination, intense exercise on the day of the examination, infection with a body temperature above 37.5 °C, use of drugs reducing body temperature, sunbathing on the day of the examination, physical therapy treatments performed, skin covered with ointments, creams, makeup or dirt, dermatological changes, tattoos in the area covered by the examination. Additionally, a suitably prepared test room was available for examined patients where the patient has to spend about 30 min before the test, because proper preparation is necessary to obtain the correct results.

The conducted research confirmed that it is important to correctly determine the area in which the average temperature value is analyzed based on the treatment plan. It was possible to observe thermal skin effects associated with radiotherapy treatment and temperature changes due to the higher dose received in each week of treatment in the studied area. It seems advantageous to analyze not only the area of PTV that obtains the highest dose, but also the areas that receive the lower dose, as in these areas we also observed a significant increase in temperature during treatment. Such observation confirmed that receiving a 10 Gy radiation dose is not indifferent to the organism, as the temperature increase was also observed in these areas.

Patients undergoing radiation therapy are monitored by the attending physician. This method of treatment is painless for patients, but may have side effects. In the case of the breast, the most common symptoms are radiation reaction. As a result of radiation, many morphological and functional disorders occur. Due to the time of appearance of the reaction, we can divide it into an early one, which occurs already during radiotherapy, or a late one, which occurs several months after the end of treatment. The first symptom of its occurrence is skin flushing. This is due to release of toxic substances that cause swelling and discoloration of the skin due to dilation of the capillaries and increased permeability. Stronger discoloration is caused by the migration of melanocytes to the surface layer and damage to the epithelium of the hair follicle lining. After delivery of a total dose of 20–25 Gy (i.e., around the third week of treatment), most patients develop a visible radiation reaction. Additionally, there may be dry peeling of the epidermis (visible as peeling of the skin and described by patients as an uncomfortable itching sensation), or moist exfoliation (serous discharge and painful exposure of the underlying dermis) [47,48]. Early radiation reactions should resolve spontaneously, but acute radiation reactions may occur in some patients; therefore, frequent monitoring of the radiation reaction is extremely important. Until now, in clinical practice, apart from an interview and visual assessment of the treatment site, no objective methods have been used to determine the area. They are classified by the physician on the basis of his experience according to the NCI CTCAE scale, where slight redness is defined as NCI CTCAE grade I; in the case of skin burns, grade III-IV NCI CTCAE; and in the case of death, the V NCI CTCAE value [49]. With the duration of treatment and the tissues receiving a higher cumulative radiation dose, the temperature asymmetry between the healthy and the treated area is greater [50,51]. Radiation reactions occurred in all patients in the PTV area that received the highest dose. It seems that the occurrence of a reaction outside this area may draw attention to the accuracy of the implementation of the treatment plan and correct any errors.

The method proposed by the authors allows for the observation of temperature changes under the influence of radiation, as well as the determination of its range and evaluation over time.

It should be noted that the highest increase in temperature occurred in the third week of treatment, when most of the examined patients developed a skin-reddening reaction at that time. However, since thermovision is non-invasive, it can be repeated many times without harm to the patient.

## 5. Conclusions

The temperature changes during radiotherapy, so the absorbed energy produces a derivative of the temperature distribution on the body surface. It is not possible to evaluate the effectiveness of radiotherapy, but indirectly obtained energy through dose distribution based on isotherms.

Moreover, the highest increase in PTV temperature was obtained in the third week of treatment, and the temperature change was 0.78 °C for the PTV area.

Results obtained during the research confirm the usefulness of the infrared thermography method in assessing the patient’s thermal response to the dose received in radiotherapy. The observations may be used to evaluate the skin reaction from radiation; however, they require further studies and new quantitative parameters.

## Figures and Tables

**Table 1:** Temperature change in PTV area for 10 exemplary patients, difference between the third week and mean temperature before radiotherapy.

	Before RT (°C)	1 Week (°C)	2 Week (°C)	3 Week (°C)	4 Week (°C)	Differences between the 3rd Week of Treatment and before RT (°C)
patient 1	33	33.4	34	34.9	34.5	1.9
patient 2	34.6	34.9	35.1	35.2	34.1	0.6
patient 3	31.7	31.8	31.9	32.4	31.7	0.7
patient 4	34.6	34.5	34.6	35.2	34.4	0.6
patient 5	34.4	34.7	34.7	35	35	0.6
patient 6	34.2	34.3	34.5	34.8	34.5	0.6
patient 7	32.7	33.8	33.8	34	33.8	1.3
patient 8	34.4	34.9	35	35.5	34.8	1.1
patient 9	34.2	34	34.1	34.8	34.1	0.6
patient 10	33.7	34	34	34.4	33.7	0.7

**Table 2:** Temperature increase in selected areas (PTV 30 Gy, 20 Gy and 10 Gy) in the third week of treatment.

	PTV	30 Gy	20 Gy	10 Gy
rise of temperature	0.78 °C	0.63 °C	0.57 °C	0.53 °C

## Data Availability

Not applicable.

## References

[1] Kopans D.B. (1984). “Early” breast cancer detection using techniques other than mammography. AJR.

[2] Kocaöz S., Özçelik H., Talas M., Akkaya F., Özkul F., Kurtuluş A., Ünlü F. (2018). The Effect of Education on the Early Diagnosis of Breast and Cervix Cancer on the Women’s Attitudes and Behaviors Regarding Participating in Screening Programs. J. Cancer Educ..

[3] Coleman C. (2017). Early Detection And Screening For Breast Cancer. Semin. Oncol. Nurs..

[4] Niell L., Freer E., Weinfurtner R., Arleo E., Drukteinis J. (2017). Screening for Breast Cancer Bethany. Radiol. Clin. N. Am..

[5] Gamagami P. (1986). Atlas of Mammography: New Early Signs in Breast Cancer.

[6] Śniadecki M. (2015). Kryteria Rozpoznawania I Wczesne Objawy Chorób Nowotworowych.

[7] Ślosarek K. (2007). Podstawy planowania leczenia w radioterapii. Polskie Towarzystwo Onkologiczne.

[8] Malicki J., Ślosarek K. (2016). Planowanie Leczenia i Dozymetria w Radioterapii.

[9] Waligórski M., Lesiak J. (2000). Podstawy Radioterapii.

[10] Łobodziec: W. (1999). Dozymetria Promieniowania Jonizującego w Radioterapii, Wyd.

[11] Kukołowicz: P. (2001). Charakterystyka Wiązek Terapeutycznych Fotonów i Elektronów.

[12] Niwińska A., Gałecki J. (2016). Current indications and methods of postoperative radiation therapy—Repetition before the exam. Oncol. Clin. Pract..

[13] Censabella S., Claes S., Orlandini M., Braekers R., Thijs H., Bulens P. (2014). Retrospective study of radiotherapy-induced skin reactions in breast cancer patients: Reduced incidence of moist desquamation with a hydroactive colloid gel versus dexpanthenol. Eur. J. Oncol. Nurs..

[14] Harper J.L., Franklin L.E., Jenrette J.M., Aguero E.G. (2004). Skin Toxicity During Breast Irradiation: Pathophysiology and Management. South. Med. J..

[15] Hymes S.R., Strom E.A., Fife C. (2006). Radiation dermatitis: Clinical presentation. pathophysiology and treatment 2006. J. Am. Acad. Dermatol..

[16] Trotti A., Bentzen S.M. (2004). The need for adverse effects reporting standards in oncology clinical trials. J. Clin. Oncol..

[17] Ellis L.M., Fidler I.J. (1995). Angiogenesis and breast cancer metastasis. Lancet.

[18] Rodenberg D.A., Chaet M.S., Bass R.C. (1995). Nitric Oxide: An Overview. Am. J. Surg..

[19] Nakamura Y., Yasuoka H. (2006). Nitric Oxide in Breast Cancer. Clin. Cancer Res..

[20] Anbar M. (1994). Hyperthermia of the cancerous breast: Analysis of mechanism. Cancer Lett..

[21] Fox S.B., Generali D.G. (2007). Breast tumour angiogenesis. Breast Cancer Res..

[22] Bauer J., Hurnik P., Zdziarski J., Mielczarek W., Podbielska H. (1997). Thermovision and its applications in medicine. Acta Bio-Opt. Inf. Med..

[23] Ring E.F.J. (1998). Progress in the measurement of human body temperature. IEEE Eng. Med. Biol. Mag..

[24] Ring E.F.J., Ammer K. (2000). The Technique of Infrared Imaging in Medicine. Thermol. Int..

[25] Ring E.F.J., Ammer K. (2012). Infrared thermal imaging in medicine. Physiol. Meas..

[26] Cholewka A., Kajewska J., Kawecki M., Sieroń-Stołtny K., Stanek A. (2017). How to use thermal imaging in venous insufficiency?. J. Therm. Anal. Calorim..

[27] Cholewka A., Stanek A., Klimas A., Sieroń A., Drzazga Z. (2014). Thermal imaging application in chronic venous disease: Pilot study. J. Therm. Anal. Calorim..

[28] Cholewka A., Stanek A., Sieroń A., Drzazga Z. (2012). Thermography study of skin response due to whole-body cryotherapy. Ski. Res. Technol..

[29] Mouli P.E.C., Kumar S.M., Senthil B., Parthiban S., Malarvizhi A.E., Karthik R. (2012). Application of Thermography in Dentistry—A Review. J. Dent. Med. Sci..

[30] Helmy A., Holdmann M., Rizkalla M. (2008). Application of Thermography for Non-Invasive Diagnosis of Thyroid Gland Disease. IEEE Trans. Biomed. Eng..

[31] Keyserlingk J.R., Ahlgren P.D. (1998). Infrared imaging of the breast; initial reappraisal using high—resolution digital technology in 100 successive cases of stage 1 and 2 breast cancer. Breast J..

[32] Herman C., Cetingul M.P. (2011). Quantitative visualization and detection of skin cancer using dynamic thermal imaging. J. Vis. Exp..

[33] Head J.F., Wang R.E. (1993). Breast thermography is a noninvasive prognostic procedure that predicts tumor growth rate in breast cancer patients. Ann. N. Y. Acad. Sci..

[34] Kennedy D.A., Lee T., Seely D. (2009). A Comparative review of thermography as a breast screening technique. Integr. Cancer Ther..

[35] Morales-Cervantes A., Kolosovas-Machuca E.S., Guevara E., Reducindo M.M., Hernández A.B.B., García M.R., González F.J. (2018). An automated method for the evaluation of breast cancer using infrared thermography. EXCLI J..

[36] Arora N., Martins D., Ruggerio D., Tousimis E., Swistel A., Osborne M., Simmons R. (2008). Effectiveness of a noninvasive digitalinfrared thermal imaging system in the detection of breast cancer. Am. J. Surg..

[37] Rassiwala M., Mathur P., Mathur R., Farid K., Shukla S., Gupta P., Jain B. (2014). Evaluation of digital infraered thermal imaging asan adjunctive screening method for breast carcinoma: A pilot study. Int. J. Surg..

[38] Ng E.Y.K. (2009). A review of thermography as promising non-invasive detection modality for breast tumors. Int. J..

[39] Spitalier H., Amalric R. (1982). Does infrared thermography truly have a role in present day breast cancer management. Biomed. Thermol..

[40] Jones C.H., Parsons C.A. (1983). Thermography of the female breast. Diagnosis of Breast Disease.

[41] Head J.F., Lipari C.A., Elliot R.L. Comparison of mammography and breast infrared imaging: Sensitivity. specificity. false negatives. false positives, positive predictive value. and negative predictive value. Proceedings of the First Joint BMES/EMBS Conference. 1999 IEEE Engineering in Medicine and Biology 21st Annual Conference and the 1999 Annual Fall Meeting of the Biomedical Engineering Society.

[42] Maillot O., Leduc N., Atallah V., Escarmant P., Petit A., Belhomme S., Sargos P., Vinh-Hung V. (2017). Evaluation of acute skin toxicity of breast radiotherapy using thermography: Results of a prospective single-centre trial. Cancer/Radiothérapie.

[43] Chan R.J., Larsen E., Chan P. (2012). Re-examining the Evidence in Radiation Dermatitis Management Literature: An Overview and a Critical Appraisal of Systematic Reviews. Int. J. Radiat. Oncol. Biol. Phys..

[44] Zhu W., Jia L., Chen G., Li X., Meng X., Xing L., Zhao H. (2019). Relationships between the changes of skin temperature and radiation skin injury. Int. J. Hyperth..

[45] Plaza D., Baic A., Lange B., Stanek A., Ślosarek K., Kowalczyk A., Cholewka A. (2021). Correlation between Isotherms and Isodoses in Breast Cancer Radiotherapy—First Study. Int. J. Environ. Res. Public Health.

[46] Ammer K. (2008). The Glamorgan Protocol for recording and evaluation of thermal images of the human body. Thermol. Int..

[47] Sanchis A.G., González L.B., Carazo J.L.S., Partearroyo J.C.G., Martínez A.E., González A.V., Torrecilla J.L.L. (2017). Evaluation of acute skin toxicity in breast radiotherapy with a new quantitative approach. Radiother. Oncol..

[48] Carmen L., Ambrosone C.B., Kropp S., Helmbold I., Schmezer P., von Fournier D., Haase W., Sautter-Bihl M.L., Wenz F. (2007). Chang-Claude, J. Predictive factors for late normal tissue complications following radiotherapy for breast cancer. Breast Cancer Res. Treat..

[49] Huang C.J., Hou M.F., Luo K.H., Wei S.Y., Huang M.Y., Su S.J., Kuo H.Y., Yuan S.S.F., Chen G.S., Hu S.C.S. (2015). RTOG, CTCAE and WHO criteria for acute radiation dermatitis correlate with cutaneous blood flow measurements. Breast.

[50] Uematsu S. (1985). Symmetry of skin temperature: Comparing one side of the body to the other. J. Am. Acad. Thermol..

[51] Niu H.H., Lui P.W., Hu J.S., Ting C.K., Yin Y.C., Lo Y.L., Liu L., Lee T.Y. (2001). Thermal symmetry of skin temperature: Normativedata of normal subjects in Taiwan. Chin. Med. J..

